# Accuracy analysis of different dose calculation algorithms for locally advanced pancreatic cancer stereotactic body radiotherapy

**DOI:** 10.7150/jca.87596

**Published:** 2023-09-04

**Authors:** Yongchun Song, Xuyao Yu, Yuwen Wang, Yang Dong, Zhiyong Yuan

**Affiliations:** 1 Department of Radiation Oncology, Tianjin Medical University Cancer Institute and Hospital, National Clinical Research Center for Cancer, Tianjin's Clinical Research Center for Cancer, Key Laboratory of Cancer Prevention and Therapy, Tianjin, 300060, People's Republic of China.; 2 Tianjin Medical University, Tianjin, 300070, People's Republic of China.; 3 Department of Radiotherapy, Tianjin Cancer Hospital Airport Hospital, Tianjin, 300308, People's Republic of China.

**Keywords:** Locally advanced pancreatic cancer, SBRT, Raytracing, Fine size pencil beam, Monte carlo

## Abstract

**Background:** The dose distribution in different optimization algorithm plans of stereotactic radiotherapy (SBRT) for locally advanced pancreatic cancer (LAPC) were compared and analyzed using monte carlo dose calculate algorithm (MC).

**Methods:** A retrospective study analyzed 26 LAPC patients treated with SBRT. The SBRT plans were designed by raytracing (RT) and fine size pencil beam (FSPB) algorithms in the CyberKnife (CK) precision system, all of which met the requirements of clinical target dose and organ at risk (OAR). Keeping the original optimization parameters unchanged, the RT and FSPB algorithm plans were recalculated by MC algorithm. The accuracy of different algorithm plnas were compared and analyzed by using planning parameters and dose distribution.

**Results:** There was no significant differences in the coverage and conformal index (CI) of the planned target volume (PTV) between RT and FSPB algorithm plans, but dose distribution of organ at risk (OAR) and the maximum dose outside the PTV boundary of 2 cm (D2cm) were lower in FSPB plans compared to RT plans, and this difference was statistically significant with p-values < 0.05. Compared to the MC algorithm, both RT algorithm and FSPB algorithm overestimated dose of the PTV and OAR. The RT algorithm was more consistent with the MC algorithm than the FSPB algorithm. The relative error of PTV coverage within the RT algorithm was 8.02% ± 1.53%, and the relative error range of OAR dose parameters was 3.32% -12.73%.

**Conclusion:** Although the FSPB algorithm could achieve rapid dose drop-off around the PTV and lower dose distribution in the OAR for pancreatic cancer SBRT plans, the algorithm error were higher than the RT algorithm. RT and FSPB algorithm overestimated the dose in the target and OAR. That was important to evaluate the clinical plans.

## Introduction

Pancreatic cancer is one of the common malignant tumors of the digestive tract, characterized by high malignancy and early metastasize. Because the lesions are often close to the stomach, duodenum, small intestine, etc., surgical treatment is difficult. More than 40% of patients experience local recurrence after surgery 1. Studies by Moningi et al. show that after radical resection of pancreatic cancer combined with radiotherapy, the local recurrence rate in the target area is low, but a small number of patients experience recurrence in the radiotherapy field and surrounding region. This requires accurate analysis of the range and dose of the target area 2. Compared with conventional radiotherapy, high-precision stereotactic body radiation therapy (SBRT) has higher geometric accuracy and dose distribution consistency. It can achieve single high-dose irradiation of tumors, increasing the lethality of tumor cells. International consensus guidelines recommend SBRT as a new treatment modality for pancreatic cancer 3.

We have found that the raytracing (RT) algorithm and the finite size pencil beam (FSPB) algorithm have large errors in calculating the scattering of beams passing through low-density tissue 4. The Monte Carlo (MC) algorithm can achieve accurate modeling of the interaction between beams and human tissues, reducing the impact of low-density cavities and surrounding osteolytic structures on dose calculation. The MC algorithm is considered the most accurate dose calculation method 5-6. This paper compares the SBRT plans for pancreatic cancer optimized using the RT and FSPB algorithms. The MC algorithm analyzes the calculation accuracy to provide a reference for selecting clinical treatment plans.

## Materials and Methods

### Design and patients

This study retrospectively evaluated the data of 26 patients with non-metastatic and inoperable locally advanced pancreatic cancer (LAPC). They received SBRT using CK between June 2020 and January 2022, in Tianjin Medical University Cancer Institute and Hospital. Table 1 shows the patient characteristic. The inclusion criteria were:

1) Age ≥ 18 years old,

2) Karnofsky Performance Status ≥ 60,

3) No other malignancies diagnosed within 5 years,

4) Histologically and/or radiologically proven unresectable LAPC,

5) Absence of nodal and metastatic disease.

The exclusion criteria were:

1) Combined autoimmune pancreatitis,

2) Pancreatic Neuroendocrine tumor,

3) Previous abdominal radiation therapy,

4) Gastric or duodenal obstruction.

All patients were implanted with fiducial labels in or near the tumor, under the guidance of B-ultrasound one week before SBRT. The MED-TEC vacuum body pad was used for shaping and fixation. Computed tomography (CT; Philips Brilliance Big Bore CT, Netherlands) was performed with 130 Kv, 400 mAs and reconstruction slice 1.25 mm. And enhanced T1-weighted magnetic resonance imaging (MRI; Siemens Magnetom 1.5 T, Siemens AG Medical Solutions, Germany) was performed with 512×512 matrix and 1.5 mm reconstruction slice. The scanning range was from the 10th thoracic vertebra (T10) to the pelvis, and the scanning layer thickness was 1.25mm. The patients should fast for > 2h before CT and MRI to ensure consistency in the shape and volume of the stomach, following the guidelines for the definition of target areas of pancreatic cancer issued by the European Society of Radiation Oncology 7.

CT and MRI images were transmitted to the Precision system (ver1.1.1.1, Accuray, US). The clinicians delineated the gross tumor volume (GTV) and OAR according to RTOG guidelines on the CT images (window level, 40 Hounsfeld units; window width, 300 Hounsfeld unit) with MRI. The GTV was expanded isotropically by 5 mm to generate planning target volume (PTV). When PTV overlaps with OAR such as gastrointestinal tract, it is needed to be corrected and deleted the overlapping part with OAR. The outline of OAR contains liver, kidney, stomach, duodenum, small intestine and spinal cord.

The median radiation dose was 40 Gy (35-45 Gy) in 5 fractions (3-6 f). Clinical requirements mandated prescription dose covered at least 99% of the GTV and 95% of the PTV. However, when the PTV was close to OAR, PTV was adjacent to OAR, plan optimization prioritized meeting OAR dose constraints, and compromised PTV coverage to at least 90% if necessary 8. The RT algorithm of fixed collimator and FSPB algorithm of multi leaf grating collimator (MLC) were used to calculate the SBRT plans for each patient. The RT and FSPB dose calculation algorithms uses the relative election density to determine the effective depth for any beam at any point in the patient anatomy. To ensure effective plan comparison, the two plans were evaluated after normalization to the prescribed dose based on the same isodose line. Considering overall clinical treatment efficiency, a single fixed collimator was used for planning and design. Then, with the same constraints, the MC algorithm was used with 1% uncertainty to recalculate and evaluate the accuracy of the two plans.The monte carlo dose calculation algorithm uses the mass density to model energy dissipation and scale the tracks while particles move through the patient.

### Dosimetric evaluation parameters

Two different optimization algorithm plans (RT and FSPB) were compared based on:

1) Conformality index (*CI*)

*CI* = PIV/PTV (1)

where PIV refered to the volume covered by 100% of the prescribed dose,

2) Coverage of the PTV (*Coverage*),

3) Mean dose in the PTV (*D_mean_*),

4) Percentage of the maximum dose irradiated and the maximum dose planned in the area 2 cm away from the PTV boundary was *D_2cm_*.

The absolute error (*σ*) between two different optimization algorithms and MC algorithm was calculated as:

*σ* = (D_RT/FSPB_ - D_RT-MC/FSPB-MC_) / D_RT-MC/FSPB-MC_×100% (2)

where D_RT/FSPB_ and D_RT-MC/FSPB-MC_ were the dose parameter values of RT/FSPB and RT-MC/FSPB-MC algorithm plans respectively.

The dose parameters for absolute error analysis included:

1) Coverage of the PTV (*Coverage*),

2) Maximum, minimum and mean dose to the PTV (*D_max_*, *D_min_* and *D_mean_*),

3) D_2cm_.

The comparative analysis parameters of OAR included:

1) Maximum dose of spinal cord (*D_max_*),

2) Maximum dose to 0.35ml and 5ml of stomach, small intestine and duodenum tissue (*D_0.35ml_* and *D_5ml_*,),

3) Volumes receiving 5 Gy and 10 Gy in the unilateral kidney near the PTV (*V_5_* and *V_10_*),

4) Volume receiving 12 Gy in the liver (*V_12_*).

### Statistical methods

The plan parameters conformed to normal distribution, and the results were expressed in the form of mean ± standard deviation (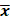
±*s*). Student* t* test was used to compare the pairwise pairwise between the planning parameters, and *p -*value < 0.05 was considered as statistically significant.

## Results

### Differences between RT and FSPB plans

Parameters of the RT and FSPB plans for pancreatic cancer in this study are shown in Tables 2 and 3. According to Table 1, there were no significant differences in the average dose (*D_mean_*) and coverage of the two planned target volumes. *D_2cm_* was lower in the FSPB plan, indicating better dose drop-off around the PTV. Because the FSPB algorithm in the Precision system used 26 pairs of multi-leaf collimator (MLC) blades for optimization, it could better shape irregular targets. As a result, the *CI* of the FSPB plans was better than the RT plans with a fixed collimator. In addition, the total number of machine hops and treatment time planned using the FSPB algorithm were significantly lower than with the RT algorithm.

According to Table 2, the *D_5ml_* of small intestine, stomach, duodenum and kidney and the *D_0.35ml_* of spinal cord in FSPB algorithm plans were lower than RT plans. That meant the SBRT plan for pancreatic cancer designed by FSPB algorithm with the MLC mode could better protect OAR around the PTV. This was consistent with the dose drop of FSPB plan in Table 1, which proves again that FSPB algorithm plans with MLC mode had the advantage of rapid convergence of dose around the target area. Because the PTV of pancreatic cancer was close to the small intestine, stomach and duodenum, FSPB algorithm plans also had no advantage in the protection of high-dose OAR (*D_0.35ml_*).

### Absolute error between two algorithms and MC algorithm

The accuracy of RT and FSPB algorithm plans was analyzed by MC algorithm. Table 4 and Table 5 show the comparison results of RT and FSPB plans for 26 LAPC. According to Table 4 and Table 5, dose calculation was higher with the RT and FSPB algorithms than the MC algorithm. RT and FSPB algorithms overestimated the dose to the PTV and OAR. The relative error between the RT algorithm plans and MC algorithm plans was less than with the FSPB algorithm plans.

Table 4 shows that the relative error in PTV coverage in the RT plans was 8.02 ± 1.53, which was better than in the FSPB algorithm plans (11.18 ± 2.76). The relative error in the PTV dose was less in the RT plans than in the FSPB algorithm plans. The relative error was lower in the high-dose area (*D_max_* and *D_mean_*) than in the low-dose area (*D_min_* and *D_2cm_*) of the PTV for both algorithms. Table 5 also shows that OAR dose parameters were higher in both algorithm plans than in the MC algorithm plans. The relative error range was 3.32%-12.73% in the RT algorithm plans and 3.74%-14.45% in the FSPB algorithm plans. The relative error was lowest for the spinal cord (*D_max_*). The relative error was highest for *V_5_* of the unilateral kidney adjacent to the PTV. This was because the PTV of pancreatic cancer patients was far from the spinal cord, and the dose to the spinal cord was usually less than 5 Gy, resulting in the lowest calculation error. Dose calculation accuracy was poor for the unilateral kidney adjacent to the target area because the radiation passed through cavities and renal parenchyma.

**Figure 1:**
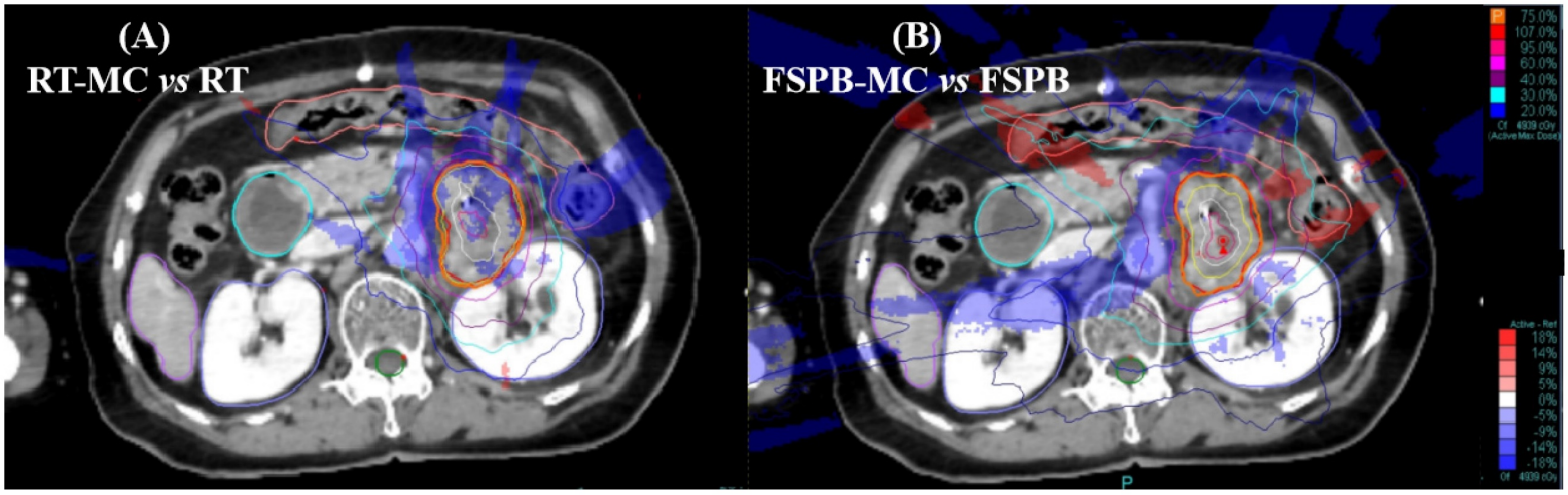
Dose distribution and difference map for a pancreatic lesion treated on Cyberknife using the Synchrony tracking system, (A) Differences between RT and MC, (B) Differences between FSPB and MC. Red = PTV, Purple = Liver, Orange = Colon, Blued = Kidney(left&right), Green = Spine cord. Isodose lines represent relative dose, values of isodose lines increase in 10% increments, from 20% (blue lines) to 100% (red lines). Rendering area represents dose difference, Blue = MC < RT/FSPB, Red = MC > RT/FSPB

Fig. 1 shows a cross-sectional view of dose difference distribution between the two algorithm plans and the MC algorithm for a patient with pancreatic cancer. The calculation results around the PTV were lower with the MC algorithm than with the RT and FSPB algorithms. At the same time, the calculation results in the low-dose area (20% isodose line) around the PTV were quite different, especially in the FSPB algorithm plan.

## Discussion

Patients with pancreatic cancer have a low survival rate and are prone to local recurrence. SBRT combined treatment modalities have been recommended by guidelines published by the Australasian Gastrointestinal Trials Group (AGITG) and the Trans-Tasman Radiation Oncology Group (TROG) 3. Lischalk et al. showed that delivering 25-30 Gy over 5 fractions of segmented SBRT can significantly improve local PTV control in pancreatic cancer patients with minimal radiation toxicity 9. By comparing SBRT plans using Varian Edge and CyberKnife (CK) systems in 15 pancreatic cancer patients, Dai et al. found that PTV dose conformity and uniformity were better with Edge plans, while CK plans minimized OAR radiation dose 10. A comparative study of CK plans, spiral tomotherapy (Tomo) plans, Edge accelerator plans, Trilogy accelerator plans and Gamma Knife plans by the Stereotactic Radiotherapy Group of the Tumor Radiotherapy Branch of the Shanghai Medical Association also found that CK plans had better dose drop-off gradients 11. Therefore, this study compared the SBRT plans for pancreatic cancer from two different CK system algorithms and analyzed dose calculation accuracy.

By comparing SBRT plans using the CK system in 20 pancreatic cancer patients, this study found that RT and FSPB algorithms met clinical needs. PTV coverage and conformality were similar with no significant differences, consistent with results comparing VMAT and proton intensity-modulated plans in pancreatic cancer patients by Ding X, et al. 12. Although this study found better dose convergence near the PTV and better OAR protection with the FSPB algorithm, previous studies show RT and FSPB cannot accurately simulate secondary electron distribution in heterogeneous media 13-14. Therefore, accurately calculating dose distribution for nonuniform cavities like the stomach and duodenum using the FSPB algorithm in pancreatic cancer SBRT plans is difficult.

Pokhrel et al. used the X-ray Voxel Monte Carlo (XVMC) algorithm to evaluate SBRT plans for non-small cell lung cancer (NSCLC) based on the superposition/convolution or inhomogeneity-corrected pencil beam (Pb_het) algorithms. Preliminary results showed 75% of patients had dose calculation bias 15. Ruiz-Boiset et al. evaluated AAA and MC algorithm SBRT plans in four NSCLC patients using GATE/GEANT4 simulation code. PTV dose consistency was within 18.4% for maximum, minimum and mean doses, but the dose distribution difference was largest for the spinal cord and major vessels 16. Han Liu et al. found the doses to 95% of the central and peripheral PTVs were overestimated by 9.7% ± 5.6% and 12.0% ± 7.3% in FP algorithm compared to the MC algorithm, through retrospective analysis seventy lung cancer patients (35 central and 35 peripheral) treated with IMRT 17. This aligns with this study's finding that dose calculation was higher than with MC algorithm plans.

## Conclusion

In conclusion, while SBRT plans for LAPC using CK system RT and FSPB algorithms provided good PTV coverage and met clinical needs safely, both algorithms overestimated target and OAR dose distribution compared to MC algorithm results. Although OAR doses were lower with the FSPB algorithm, its calculation errors were greater than with the RT algorithm versus MC calculation. Algorithm accuracy should therefore be further considered in clinical treatment evaluation. Adaptive SBRT may help resolve dosimetric errors from anatomical changes. This retrospective study was limited by the small sample size and lack of analysis on clinical efficiency, accuracy or outcome. We will expand the cohort in future studies would further validate our findings.

## Figures and Tables

**Table 1:** 26 Patient characteristics

	Number of patients (%)
**Age (years) (Median)**	47-74 (62)
≥ 60	15 (57.69%)
< 60	11 (42.31%)
**Sex**	
Male	12 (46.15%)
Female	14 (53.85%)
**Location of tumor**	
Head	10 (38.46%)
Neck	2 (7.69 %)
Body	6 (23.08%)
Tail	8 (30.77%)
**Tumor volume (cm^3^)**	
< 10	3 (11.54%)
10-50	16 (61.54%)
> 50	7 (26.92%)
**Prescriotion dose (Gy) (Median)**	30-45 (40)
**Fraction (Median)**	3-6 (5)

**Table 2:** Dosimetric parameters of RT and FSPB plans in 26 patients with pancreatic cancer (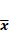
±*s*)

	RT	FSPB	*p*-value
*CI*	1.11 ± 0.05	1.08 ± 0.03	0.02
*Coverage* (%)	95.22 ± 2.75	95.91 ± 0.78	>0.05
*D_mean_* (%)	84.50 ± 3.54	85.62 ± 0.98	>0.05
*D_2cm_* (%)	48.04 ± 14.82	42.44 ± 10.95	0.03
*Total MU*	32058.53 ± 1955.42	28800.9 ± 4900.79	0.01
*Time* (min)	39.25 ± 9.81	35.54 ± 8.47	0.02

**Table 3:** Dosimetric data of OAR in RT and FSPB plans of 26 patients with pancreatic cancer** (**
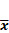
±*s***)**

		FSPB	RT	*P*-Value
Spinalcord	*D_max_* (Gy)	5.81 ± 2.57	6.68 ± 3.66	0.02
Bowel	*D_0.35ml_* (Gy)	27.47 ± 10.58	27.42 ± 10.79	0.18
	*D_5ml_* (Gy)	13.27 ± 8.25	13.53 ± 9.10	0.03
Stomach	*D_0.35ml_* (Gy)	18.07 ± 10.42	18.21 ± 12.30	0.39
	*D_5ml_* (Gy)	10.78 ± 6.57	11.14 ± 8.10	0.04
Duodenum	*D_0.35ml_* (Gy)	15.77 ± 6.71	15.98 ± 8.21	0.48
	*D_5ml_* (Gy)	4.13 ± 5.36	5.09 ± 7.50	0.02
Kidney	*V_5_* (ml)	13.51 ± 19.60	19.07 ± 27.19	0.66
	*V_10_* (ml)	3.57 ± 9.87	4.40 ± 11.67	0.03
Liver	*V_12_* (ml)	1.84 ± 2.95	3.53 ± 3.21	0.17

**Table 4:** Relative error of target dose between MC, RT and FSPB SBRT for 26 pancreatic cancer cases (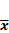
±*s*)

	*Coverage*	PTV			D_2cm_
		*D*Max	Dmean	Dmin	
RT-MC	8.02 ± 1.53	1.12 ± 0.68	2.78 ± 0.81	6.56 ± 3.47	8.79 ± 4.89
FSPB-MC	11.18 ± 2.76	3.88 ± 0.92	4.74 ± 2.12	5.35 ± 4.96	13.6 ± 9.18

**Table 5:** Relative error of OAR dose between MC, RT and FSPB SBRT for 26 pancreatic cancer cases (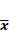
±*s*)

	Spine cord	Stomach		Duodenom	
	*D_max_*	*D0.35ml*	*D_5ml_*	*D0.35ml*	*D_5ml_*
RT-MC	3.32 ± 2.41	4.19 ± 3.72	4.59 ± 4.50	4.84 ± 4.65	6.54 ± 5.71
FSPB-MC	3.74 ± 4.56	6.53 ± 5.04	6.12 ± 2.28	8.96 ± 5.68	5.20 ± 1.55
	Lever	Kidney		Colon	
	*V_12_*	*V_5_*	*V_10_*	*D0.35ml*	*D_5ml_*
RT-MC	5.94 ± 4.39	12.73 ± 4.39	7.21 ± 14.39	6.93 ± 1.69	7.75 ± 2.12
FSPB-MC	6.31 ± 5.26	14.45 ± 7.98	9.32 ± 15.46	5.70 ± 4.17	6.80 ± 4.17

## Abbreviations

SBRT: stereotactic radiotherapy
MC: monte carlo
RT: raytracing
FSPB: fine size pencil beam
CK: CyberKnife
OAR: organ at risk
RTOG: radiation therapy oncology group
CI: conformal index
PTV: planned target volume
GTV: gross target area
KPS: karnofsky performance status
MLC: multi leaf grating collimator
*D_2cm_*: the maximum dose outside the PTV boundary of 2 cm
*D_mean_*: mean dose
*D_max_*: maximum dose
*D_min_*: minimum dose
V_5_: volumes receiving 5 Gy
V_10_: volumes receiving 10 Gy
V_12_: volumes receiving 12 Gy
